# Metallothioneins in Dental Implant Treatment Failure and Periodontitis in Patients with Down’s Syndrome: Validation of Results

**DOI:** 10.3390/genes13061028

**Published:** 2022-06-08

**Authors:** María Baus-Domínguez, Raquel Gómez-Díaz, José-Luis Gutiérrez-Pérez, Daniel Torres-Lagares, Guillermo Machuca-Portillo, María-Ángeles Serrera-Figallo

**Affiliations:** 1 Department of Dentistry, Faculty of Dentistry, University of Seville, 41009 Seville, Spain; mbaus95@gmail.com (M.B.-D.); gmachuca@us.es (G.M.-P.); maserrera@us.es (M.-Á.S.-F.); 2 Institute of Biomedicine of Seville, 41013 Seville, Spain; rgomez-ibis@us.es; 3 Oral and Maxillofacial Unit, Virgen del Rocio Hospital, 41013 Seville, Spain; jlgp@us.es; 4 Oral Surgery Department, Faculty of Dentistry, University of Seville, 41009 Seville, Spain

**Keywords:** Down’s syndrome, periodontal disease, bone biology, clinical outcomes, gene expression, validation, systemic disease

## Abstract

Metallothioneins (MTs) are low molecular weight cysteine-rich proteins that can bind up to seven zinc ions. Among their numerous functions, MTs appear to act as protectors against oxidative and inflammatory injury. In our first published study, we reported downregulation of the isoforms *MT1B* (fold distance (FD) −2. 95; *p* = 0.0024), *MT1F* (FD −1.72; *p* = 0.0276), *MT1X* (FD −3.09; *p* = 0.0021), *MT1H* (FD −2.39; *p* = 0.0018), *MT1M* (FD −2.37; *p* = 0.0092), *MT1L* (FD −2. 55; *p* = 0.0048), *MT1E* (FD −2.71; *p* = 0.0014), *MT2A* (FD −2.35; *p* = 0.0072), *MT1G* (FD −2.24; *p* = 0.0118), and *MT1A* (FD −2.82; *p* = 0.0023) by comparing Down’s syndrome patients with periodontal disease and implant failure to those without periodontal disease and with a positive progression of their implants. In this gene validation study, we intended to verify the results of our first gene expression analysis. Materials and Methods: In our retrospective case–control study, we performed retrotranscription (RT-qPCR) of 11 RNA-to-cDNA samples using the SuperScript™ VILO™ kit (50; reference 1,176,605) from Thermo Fisher. We conducted the study using the real-time PCR technique on the q-PCR ViiA 7 platform from Thermo Fisher. We chose the format of the Taqman Array Plate 16 Plus (reference 4,413,261) from Thermo Fisher, which accommodates 12 genes plus four controls (*GAPDH, 18S, ACTB,* and *HPRT1*). We conducted the analysis of the plates using the Thermo Fisher Cloud Web Software. Results: The results obtained through gene validation analysis show that in PD+RI+ patients, the genes encoding the isoforms *MT1F* (FD 0.3; *p* = 0.039), *MT1X* (FD 338; *p* = 0.0078), *MT1E* (FD 307; *p* = 0.0358), and *MT2A* (FD 252; *p* = 0.0428) continue to show downregulation, whereas *MT1B* (FD 2.75; *p* = 0.580), *MT1H* (FD 281; *p* = 0.152), *MT1L* (FD 354; *p* = 0.0965), and *MT1G* (FD 336; *p* = 0.0749) no longer show statistically significant results.

## 1. Introduction

Metallothioneins (MTs) are low molecular weight cysteine-rich proteins that can bind up to seven zinc ions. These proteins play an important role in the absorption, distribution, storage, and release of metals to maintain physiological concentrations [1,2,3]. This control by MTs is strictly necessary to avoid oxidative stress [1,3,4,5,6,7,8,9].

MT synthesis can be induced by numerous factors such as metals, chemical agents, cytokines, oxidative stress, infection, and inflammation [3,4,10]. 

Several studies have confirmed that MTs function as cytoprotective agents against oxidative and inflammatory injury because they can scavenge a wide range of reactive oxygen species, and their ability to scavenge hydroxyl radicals is 3009 times more potent than that of glutathione, the most abundant antioxidant in the cytosol [1]. Furthermore, MTs appear to inhibit certain pro-inflammatory cytokines, such as the interleukins IL-6 and IL-12, as well as the tumor necrosis factor (TNF-α), and thus may suppress inflammation [3,5]. Similarly, in vitro studies show that exo-MT can induce potent lymphocyte proliferation [4,10] and even increase it in the presence of lipopolysaccharide [10]. 

In humans, the MT genes are located at the q13 locus on chromosome 16 (16q13). Currently, up to 17 genes have been identified in this cluster, and at least 11 of them are functional. Eight correspond to MT1 isoforms (*MT1A*, *MT1B*, *MT1E*, *MT1F*, *MT1G*, *MT1H*, *MT1M*, and *MT1X*), and the others have only one functional gene, *MT2A*, *MT3*, and *MT4* [2,3,5,6,10]. 

In our first published study [11], we reported that Down’s syndrome patients diagnosed with periodontal disease and implant failure showed alterations in the gene expression (downregulation) of *MT1B*, *MT1F*, *MT1X*, *MT1H*, *MT1M*, *MT1L*, *MT1E*, *MT2A*, and *MT1G* isoforms, confirming the hypothesis that the MT1G isoforms are not functional, and the alternative hypothesis that MTs are expressed differently when comparing Down’s syndrome patients with periodontal disease and dental implant failure to those without periodontal disease and with a positive progression of their implants after two years. 

Although details regarding all the functions MTs can perform remain unknown, based on the available literature, the alteration in the expression of *MT1* and *MT2* genes is related to alterations in the early stages of bone healing that can lead not only to the failure of osseointegration of dental implants but also to the presence of low-strength bone with elevated susceptibility to periodontal disease and peri-implantitis. 

Through the present gene validation study, we wanted to verify the results we obtained from the first gene expression analysis [11].

## 2. Materials and Methods

### 2.1. Type of Study

This study was in accordance with the guidelines of the Declaration of Helsinki of the World Medical Association, Ethical Principles for Medical Research Involving Human Subjects [12]. Our retrospective case–control study approved by the corresponding Ethics Committee (Ethics Committee of the Hospital Virgen del Rocío; File PI-0081-2016). 

The study was descriptive and observational, and the only invasive procedures performed on patients were the collection of a small amount of blood and a dental examination. The patient (or the person responsible for them) gave consent based on the research’s direct benefits to the patient. 

### 2.2. Selection of Patients and Study Groups

The study groups were composed of Down’s syndrome patients diagnosed with periodontal disease (PD) at the time of the examination and failed implants (rejection of implants, RI) after two years of progression (PD+RI+) versus Down’s syndrome patients without periodontal disease and with positive implant progression after two years (PD−RI−).

The study’s exclusion criteria were patients without Down’s syndrome, patients receiving treatment that could potentially affect bone metabolism, patients treated with short or immediately loaded implants, patients with active or untreated periodontal disease, and patients with implants that had a progression period of less than two years.

In our study, we do not reflect data concerning pocket depth measurements and/or attachment loss of the remaining teeth of the patients since these data are of no interest for our study, as our variable “periodontal disease (PD)” was taken as a dichotomous variable (yes (PD+)/no (PD−)), as well as implant failure (yes (RI+)/no (RI−)).

The diagnosis of periodontal disease, as well as the evolution of the implants, was made based on the patients’ medical records and the comparison of panoramic radiographs (immediate postoperative period at two years progression) to calculate the marginal bone loss (MBL) and to differentiate whether the bone defects related to the implants were due to a placement defect or to bone loss resulting from peri-implantitis. 

Calculate the MBL of bone loss using the Lagervall and Jansson index [13] validated for its use in this type of study by Corcuera-Flores et al. [14]. This method divides the implants into four groups according to their MBL:

- Grade 0: implants without marginal bone loss.
- Grade 1: marginal bone loss of one-third or less of the total length of the implant.
- Grade 2: one-third, but less than two-thirds, of the total length of the implant.
- Grade 3: marginal bone loss greater than two-thirds of the total length of the implant.
- A fifth group (Grade 4) was added of those patients whose implant was lost.

In our study, a failed implant (RI+) is understood as lost after two years of follow-up, or peri-implant bone loss of at least grade 2 on the Lagervall and Jansson scale [13].

We retrieved demographic and clinical data from the patients’ medical records and verified all essential data.

### 2.3. Sample Collection and Total RNA Isolation

During the patients’ clinical examinations, two blood samples per subject were collected in PAXgene® tubes (reference 762,165) for RNA extraction, and one more blood sample was collected in a 3-mL VACUTTE tube with EDTA (anticoagulant) for DNA extraction.

The samples were transported to the processing center refrigerated at 2–8 °C for up to 5 days. The tubes were stored at −20 °C or −80 °C as appropriate.

RNA samples were extracted using the PAXgene™ BLOOD miRNA KIT (reference 763,134) from QIAGEN. The extraction was carried out in the QIAcube automated station of the same brand. 

Subsequently, we prepared a database of the samples, detailing, among other things, the RNA quantification data. First, we quantified RNA concentrations using a visible light spectrophotometer with the Thermo Nanodrop 2000C equipment to ensure that the samples were well processed before conservation. Second, we took a much more precise measurement—in this case, only using the samples selected for the first gene expression study—using fluorescence and the Thermo Qubit 3.0 equipment. (reference Q33,216; Singapore, Malaysia) from Invitrogen™ by Life Technologies (Thermo Fisher Scientific, Waltham, MA, USA).

### 2.4. Validation Study of Gene Expression

We performed retrotranscription (RT-qPCR) of 11 RNA-to-cDNA samples using the SuperScript™ VILO™ kit (50; reference 1,176,605; Carlsbad, CA, USA) from Invitrogen™ by Life Technologies (Thermo Fisher Scientific).

We used the SuperScript™ VILO™ kit and not the High-Capacity RNA-to-cDNA kit because of its robustness; of the two, the VILO™ kit has a much higher tolerance to inhibitors, although the kits are quite similar in terms of efficiency. In this case, working from PAXgene tubes, we chose VILO in case any residues from the tubes were present in the samples and could generate some inhibition. 

Normalization prior to RT is not mandatory, but it is recommended because even if 100% efficiency is assumed for cDNA conversion, including small amounts of RNA, it could lead to small variations in efficiency. The SuperScript™ VILO™ kit contains 4 L of the master mix, which already includes the enzyme. Thus, completing up to 20 L of final reaction with RNA (up to a maximum of 2.5 g) and water (if necessary) allows up to 16 L of RNA to be included. However, the endogenous RNA will correct a posteriori, so, as mentioned at the beginning, normalization is not obligatory, although it is always recommended. 

In our case, we normalized the samples to 18 ng/L, which is the minimum amount. By placing 16 L in each RT, we place a total of 288 ng (0.288 g) of RNA in each RT (Table 1). 

After this normalization, we quantified the cDNA to check the efficiency, so no further normalization was necessary. We quantified the 11 samples’ cDNA using a Nanodrop 2000C spectrophotometer.

The samples were kept in deep freeze at −20 °C until the end of the study. Subsequently, we stored the samples at −80 °C in case a re-extraction was needed.

We designed a table with the IDs of the assays corresponding to the gene validation study following the below steps:

- Avoid genes located in the 5′ UTR region because this is a variable region that can differ between different transcripts, which can cause analysis problems.
- Select the best coverage that detects the highest possible number of transcripts that are usually located between 2 exons, where their amplicon is usually as small as possible.
- Selection of probe spans exon assay, to avoid detection of and contamination by genomic DNA (gDNA). We analyzed only complementary DNA (cDNA).
- Selection of the smallest amplicon to ensure the best efficiency in cases of small degradation (i.e., if there are cuts in the DNA strand, the smaller the amplicon, the less likely it is that the cut is located in that region).
- Include a negative retrotranscription (RT) control (i.e., include a sample that undergoes RT but where the RT enzyme is not included in the reaction). Thus, if amplification is finally obtained in that sample, it is because gDNA is being detected.

We performed the study using the real-time PCR technique on the q-PCR ViiA 7 platform from Thermo Fisher. Following the above indications, the format chosen was the Taqman^®^ Array Plate 16 Plus (reference 4,413,261; CA, USA) from Applied Biosystems™ by Thermo Fisher Scientific, which can accommodate 12 genes and 4 controls (Table 2). Two samples can be loaded on each plate in technical triplicates, requiring a total of 6 cards to study 11 samples.

The endogenous genes to be studied were *GAPDH* (glyceraldehyde 3-phosphate dehydrogenase), *18S* (18S ribosomal RNA), *ACTB* (B-Actin), and *HPRT1* (hypoxanthine phosphoribosyltransferase 1). Three of these are enough for the Thermo Fisher Cloud Web Software from Applied Biosystems™ by Thermo Fisher Scientific (version 2021.1.1-Q1-21-build11, CA, USA) to decide which is the best.

We conducted the analysis of the plates using the Thermo Fisher Cloud Web Software.

### 2.5. Statistical Analysis 

Statistical analysis was performed using the *t*-test of independent samples (one-tailed distribution, unequal standard deviation, *p*-value < 0.05). For quantitative real-time RT-PCR, the median values of the quantification cycle (Cq) for the target genes (*MT1A*, *MT1B*, *MT1E*, *MT1F*, *MT1G*, *MT1H*, *MT1M*, *MT1X*, *MT2A*, *MT3 y MT4*) and the reference genes (*HPRT1*, *18S*, *GAPDH*, *ACTB*) were compared in both groups (PD+RI+ vs. PD−RI−). 

## 3. Results

### 3.1. Participants and Characteristics

We compared a total of 11 Spanish patients with Down’s syndrome; seven of them were not diagnosed with periodontal disease and had positive implant progression at 2 years, whereas four patients had active periodontal disease together with implant failure (Table 3).

Demographic data are not significant and do not influence the results obtained. No statistically significant differences were observed when comparing by sex, age, or any other factor other than the one taken as a reference, which is the presence or absence of periodontal disease and implant rejection or positive evolution of implants.

Due to the strict inclusion and exclusion criteria, very few patients were included in the study. In fact, the main reason for conducting a second study, gene validation analysis, based on the results obtained in our previously published study, was the small sample size with which we had worked. 

Additionally, we would like to point out that we wanted to include a third study group formed by Down’s syndrome patients not diagnosed with periodontal disease but who had failed implants; however, no patients with these characteristics were found, and this fact was also considered a result.

### 3.2. Gene Expression Analysis

The results were obtained from RNA analysis using the Affymetrix Microarray Software. We compared the blood samples from the patients with periodontal disease and negative implant evolution (PD+RI+) to those from the patients without periodontal disease and positive implant evolution (PD−RI−), revealing among the former the low expression of genes encoding isoforms *MT1B* (FD −2. 95; *p* = 0.0024), *MT1F* (FD −1.72; *p* = 0.0276), *MT1X* (FD −3.09; *p* = 0.0021), *MT1H* (FD −2.39; *p* = 0.0018), *MT1M* (FD −2.37; *p* = 0.0092), *MT1L* (FD) −2. 55; *p* = 0.0048), *MT1E* (fold change; FD −2.71; *p* = 0.0014), *MT2A* (FD) −2.35; *p* = 0.0072), *MT1G* (FD −2.24; *p* = 0.0118), and *MT1A* (FD −2.82; *p* = 0.0023). 

However, *MT3* and *MT4* isoforms did not show significantly altered expression.

### 3.3. Gene Validation Analysis

**Figure 1:**
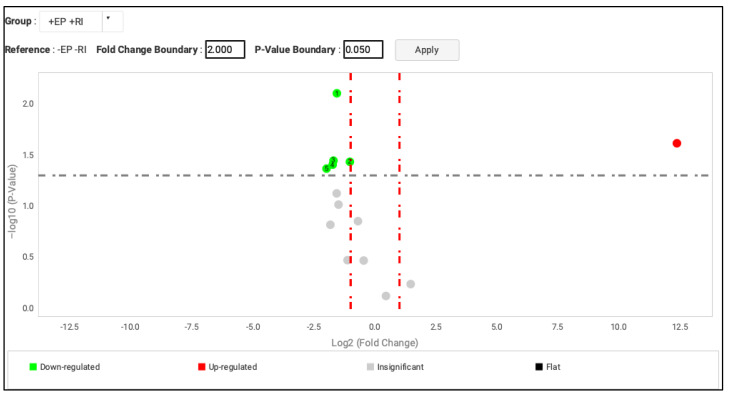
Volcano plot thermo image. Comparison of the results PD+RI+ vs. PD−RI− (statistical analysis using the *t*-test; fold change boundary: 2.000; *p*-value boundary: 0.05. *MT1F* ^4^, *MT1X* ^1^, *MT1E* ^3^, *MT2A* ^5^ (downregulated genes), and *HPTR1* ^2^ (endogenous gen) are shown in green. The *MT1M* gene is highlighted in red. PD = EP (Spanish acronyms for periodontal disease).

The results obtained through gene validation analysis, using the *t*-test, show that in PD+RI+ patients, the genes encoding the isoforms *MT1F* (FD 0.3; *p* = 0.039), *MT1X* (FD 338; *p* = 0.0078), *MT1E* (fold change; FD 307; *p* = 0.0358), and *MT2A* (FD 252; *p* = 0.0428) continued to show downregulation, whereas *MT1B* (FD 2.75; *p* = 0.580), *MT1H* (FD 281; *p* = 0.152), *MT1L* (FD 354; *p* = 0.0965), and *MT1G* (FD 336; *p* = 0.0749) no longer showed statistically significant results (Table 4) (Figure 1).

Interestingly, we observed the *MT1M* gene (FD 5,273,946; *p* = 0.0241) to be overexpressed in this analysis (Table 4) (Figure 1).

**Figure 2:**
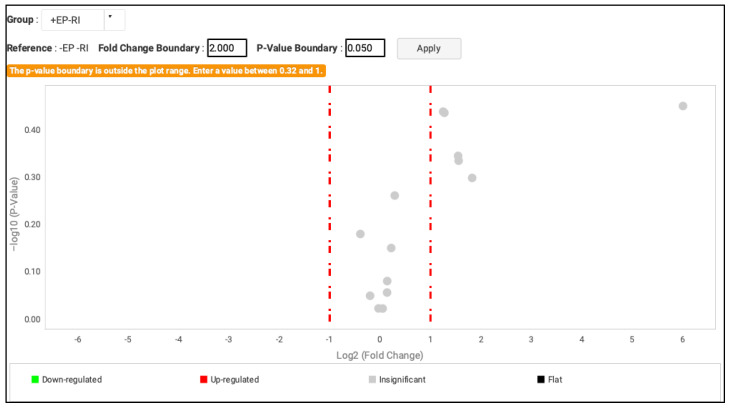
Volcano plot thermo image. Comparison of the results PD+RI− VS PD−RI− (statistical analysis using the *t*-test; fold change boundary: 2.000; *p*-value boundary: 0.05). All the isotherms studied, *MT1F* (FD 1.036; *p* = 0.9495), *MT1X* (FD 1.103; *p* = 0.8305), *MT1E* (FD 2.421; *p* = 0.36544), and *MT2A* (FD 0.762; *p* = 0.6600), *MT1B* (FD 3.549; *p* = 0.5021), *MT1H* (FD 2.945; *p* = 0.4616), *MT1L* (FD 1.100; *p* = 0.8783), and *MT1G* (FD 2.380; *p* = 0.3633) showed statistically significant results. PD = EP (Spanish acronyms for periodontal disease).

In contrast, the gene validation analysis does not show statistically significant results when performed on the PD−RI− or PD+RI− sample groups (Figure 2).

## 4. Discussion

The present validation study confirmed that the genes for the *MT1F*, *MT1X*, *MT1M*, *MT1E*, and *MT2A* isoforms exhibit a statistically significant alteration of expression in Down’s syndrome patients with active periodontal disease and implant failure. 

We recall that the research’s aim was to find out whether there are statistically significant differences in gene expression that could explain the different clinical situations at the oral level with respect to the progression of dental implants in patients suffering from the same systemic condition, chromosome 21 trisomy. 

The results of altered MT expression that were first published came from the comparison between Down’s syndrome patients with periodontal disease and implant failure (PD+RI+) after two years of progression versus Down’s syndrome patients without periodontal disease and with a positive progression of their implants (PD−RI−). However, when we compared Down’s syndrome patients with periodontal disease and implant failure (PD+RI+) to those with periodontal disease and a positive progression of their implants (PD+RI−), we observed no significantly statistical alterations in MT expression. This leads us to believe that when matching the periodontal disease variable, it is other genes and not the MT genes that are responsible for the variability in clinical presentation in terms of periodontal disease and dental implant failure, as published in one of our previous studies [15]. 

This hypothesis is supported by the fact that if the patient group is modified again to compare Down’s syndrome patients with periodontal disease and positive implant progression (PD+RI−) to those without periodontal disease and with positive implant pr (PD−RI−), the MTs appear again with altered gene expression, downregulated for the PD+RI− group (Table 5).

It should also be noted that no patients were found who did not have periodontal disease but had implant failure, which we also consider to be an important result. This could be explained by the hypothetical etiopathogenesis shared by both diseases. As is well known, periodontitis is defined as a multifactorial chronic inflammatory disease induced by periodontal pathogens whose sustained inflammation over time leads to the loss of tooth attachment through damage to the periodontium itself, loss of alveolar bone, and, ultimately, tooth loss. Periodontal disease has been linked to multiple systemic inflammatory diseases that share the same mechanisms at the molecular level (increased levels of pro-inflammatory cytokines) that would result in “systemic inflammation” [16,17,18,19,20]. These locally and systemically increasing inflammatory mediators, together with the virulence factors of periodontal pathogens, can lead to rapid peri-implant bone loss leading to dental implant loss. Therefore, if the patient does not have periodontal disease and, therefore, is assumed that his systemic inflammatory condition is within the limits considered nonpathogenic, a positive evolution of his implants can be expected, or in other words, failure of the dental implants due to a genetic inflammatory condition is not observed since the control of systemic inflammatory disorders leads to an improvement at the periodontal and peri-implant level showing antiinflammatory and antioxidant activities on the one hand, and stimulating properties that promote endothelial function, angiogenesis, and bone formation on the other [16].

Whereas the results for *MT1F*, *MT1X*, *MT1E*, and *MT2A* correlate with those obtained in the first instance in the previously published gene expression study [11], showing downregulated gene expression, *MT1M* shows a statistically significant altered expression but with a surprising result: in the validation, it was shown to be overexpressed, whereas in the gene expression study [11], it was observed to be downregulated. 

Despite the scarce literature on MTs’ functions, it has already been hypothesized that MTs could play an important role in implant osseointegration because MTs are important protectors against oxidative stress [1,3,4,5,6,7,8,10], which has been linked to the inhibition of osteoblast cell differentiation [20,21].

Similarly, another study [22], more specifically, related *MT1* and *MT2* isoforms to an important role during the early stages of osteoblastic cell differentiation from mesenchymal stromal stem cells. 

In contrast, as a source of zinc and copper, MTs are activators of several metalloenzymes, including alkaline phosphatase [5], an enzyme that appears to participate in the regulation of the proliferation, migration, and differentiation of osteoblastic cells, in addition to facilitating osteoid mineralization.

As discussed above, strict inclusion and exclusion criteria severely limited patient participation in this study. The small sample size is the main reason for the present gene validation study. Therefore, since our results in our first gene expression study [11] were positive and we decided to validate them, and since we have worked with good relevance criteria, and statistical significance levels below 0.05 (*p*-value) have again obtained satisfactory results, we do not consider it appropriate to increase the sample size. This is something we would have considered if the validation results had been negative.

On the other hand, our validated results may serve to guide new avenues of research into the failure of dental implants in Down’s syndrome patients, and, in the not too distant future, the predictability of dental implant treatment prior to implant surgery may be known. 

## 5. Conclusions

Although it is true that there remains much to study and understand about MTs and their role, and although we are only hypothesizing now, it is clear from this gene validation study that this difference in the degree of gene expression of at least the *MT1F*, *MT1X*, *MT1E*, and *MT2A* isoforms could partly explain not necessarily the failure of implants in this type of patient, but rather the complex gene network behind the implant failure process. This study also clarified that this gene is indeed involved in different metabolic pathways leading to the clinical situation PD+RI+, given that we have obtained satisfactory and confirmatory results despite the small cohort of patients with whom we worked.

However, much remains to be known about the etiopathogenesis of peri-implantitis and its relationship to periodontal disease. Although there are currently numerous studies that relate periodontitis to systemic disorders and their bidirectional improvements, in relation to the inflammatory state of the patient, when treating periodontal disease and/or a systemic condition, in certain cases, improvements in peri-implantitis are not observed.

The current lack of studies on peri-implantitis in patients with Down’s syndrome highlights the need for further research on the subject that could facilitate the path of dental rehabilitation in this type of patient, as in certain circumstances it is the only available therapeutic option. Our validation study aims to clarify the complex gene network behind implant failure in these patients in order to be able to direct dental treatment and predict the response to periodontal and implant therapy in the future.

## Figures and Tables

**Table 1:** Quantification and normalization of RNA per sample to 288 ng total at 16 µL RT.

Sample RNA	Processing	Nucleic Acid Conc.	Unit	260/280	Sample Type	Factor	Vol. Final Sample	QUBIT 3.0	Unit	Normalized 0.288 ngr Total in 16 µL of RT	Biogroup
EDCS 1	19/1/18	27.9	ng/µL	2.71	RNA	40	50 µL	44,800.00	ng/µL	6.43	PD+RI+
EDCS 4	19/1/18	19.6	ng/µL	3.01	RNA	40	50 µL	26.600	ng/µL	10.83	PD−RI−
EDCS 10	19/1/18	34.8	ng/µL	2.60	RNA	40	50 µL	45.200	ng/µL	6.37	PD−RI−
EDCS 11	8/3/18	21.3	ng/µL	3.62	RNA	40	50 µL	26.000	ng/µL	11.08	PD+RI+
EDCS 13	8/3/18	35.4	ng/µL	2.88	RNA	40	50 µL	37.200	ng/µL	7.74	PD−RI−
EDCS 16	18/1/18	17.1	ng/µL	3.26	RNA	40	50 µL	66.600	ng/µL	4.32	PD+RI+
EDCS 33	19/1/18	65.4	ng/µL	2.30	RNA	40	50 µL	18.460	ng/µL	15.60	PD−RI−
EDCS 34	18/1/18	62.5	ng/µL	2.36	RNA	40	50 µL	45.000	ng/µL	6.40	PD−RI−
EDCS 35	18/1/18	68.0	ng/µL	2.34	RNA	40	50 µL	37.800	ng/µL	7.62	PD−RI−
EDCS 36	19/1/18	35.6	ng/µL	2.56	RNA	40	50 µL	40.200	ng/µL	7.16	PD−RI−
EDCS 51	19/1/18	61.9	ng/µL	2.35	RNA	40	50 µL	55.800	ng/µL	5.16	PD+RI+

**Table 2:** Design of each plate with the genes to be studied and the controls. We worked with *GAPDH, 18S,* and *HPRT1* because these are the endogenous genes that have been used in similar patients in other publications. Because there were two gaps left on the plate, we decided to introduce the endogenous B-Actin and repeat the *MT1F* to ensure that it was not downregulated.

ID Assay Thermo	ID Gene	HGNC ID (Gene)	Approved Symbol	Approved Name	Previous Symbols	Synonyms	Chromosome
Hs01591331_g1 Detect Genomic DNA. Best Coverage	TC1600007962	HGNC:7393	MT1A	metallothionein 1A	MT1, MT1S		16q13
Hs00538861_m1 Probe spans exons	TC1600007964	HGNC:7394	MT1B	metallothionein 1B	MT1, MT1Q		16q13
Hs01938284_g1 Detect Genomic DNA. Best Coverage	TC1600007959	HGNC:7397	MT1E	metallothionein 1E	MT1	MTD	16q13
Hs00744661_sH Detect Genomic DNA. Best Coverage	TC1600007965	HGNC:7398	MT1F	metallothionein 1F	MT1		16q13
Hs02578922_gH Smallest amplicon	TC1600010421	HGNC:7399	MT1G	metallothionein 1G	MT1	MT1K	16q13
Hs00823168_g1 Detect genomic DNA. Best Coverage	TC1600007966	HGNC:7400	MT1H	metallothionein 1H	MT1		16q13
Hs00828387_g1 Detect genomic DNA. Probe spans exons	TC1600007960	HGNC:14296	MT1M	metallothionein 1M	MT1, MT1K		16q13
Hs00745167_sH Detect genomic DNA. Best coverage	TC1600011399	HGNC:7405	MT1X	metallothionein 1X	MT1	MT-1l	16q13
Hs02379661_g1 Best coverage	TC1600007957	HGNC:7406	MT2A	metallothionein 2A	MT2		16q13
Hs01921768_s1 Best coverage	TC1600007955	HGNC:7408	MT3	metallothionein 3		GIF	16q13
Hs00262914_m1 Probe spans exons. Best coverage	TC1600007953	HGNC:18705	MT4	metallothionein 4		MTIV	16q13
Hs99999909_m1/			HPRT1	Hypoxanthine Phosphoribosyltransferase 1			
Hs99999901_s1			18S	Eukaryotic 18S rRNA			
Hs99999905_m1			GAPDH	glyceraldehyde-3-phosphate dehydrogenase			
Hs99999903_m1			ACTB	Actin B			
Hs00744661_sH Detect Genomic DNA. Best coverage	TC1600007965	HGNC:7398	MT1F	metallothionein 1F	*MT1*		*16q13*

**Table 3:** Clinical data of the patients included in the study. To increase the number of patients in the control group, we included Patients 9, 10, and 11, who did not yet have implants in place. However, these patients did not have periodontal disease and would therefore not be candidates for early failure after implant placement.

Patient	Group	Age (Years)	Sex	Smoker	Drinker	History of Controlled Periodontal Disease	Implants Placed	Bone Regeneration	Result at Two Years Follow-Up
1	PD+RI+	41	F	No	No	Yes	2	No	1 implant lost and 1 implant with severe peri-implantitis
2	PD+RI+	39	F	No	No	Yes	3	No	1 implant lost and 2 implants with severe peri-implantitis
3	PD+RI+	33	M	No	No	Yes	4	No	2 implants with severe peri-implantitis
4	PD+RI+	35	M	No	No	Yes	12	No	3 implants lost
5	PD−RI−	40	F	No	No	No	3	No	No implant failure or peri-implantitis
6	PD−RI−	34	F	No	No	No	2	No	No implant failure or peri-implantitis
7	PD−RI−	43	F	No	No	No	3	No	No implant failure or peri-implantitis
8	PD−RI−	48	F	No	No	No	2	No	No implant failure or peri-implantitis
9	PD−RI−	44	M	No	No	No	0	No	No implant failure or peri-implantitis
10	PD−RI−	38	M	No	No	No	0	No	No implant failure or peri-implantitis
11	PD−RI−	44	M	No	No	No	0	No	No implant failure or peri-implantitis

**Table 4:** Comparison of the results obtained from the gene validation analysis and the first results obtained on Affymetrix Microarrays (statistical analysis using the *t*-test; fold change boundary: 2.0; *p*-value boundary: 0.05).

Target	Validation Fold Change	Validation *p*-Value	Number of Volcano Plot Thermo Image (Figure 1)	Validation Result	Affymetrix Result
Hs00262914_m1 MT4	1.361	0.758139	Insignificant	Insignificant	Insignificant
Hs00538861_m1 MT1B	2.75	0.58000445	Insignificant	Insignificant	Downregulated
Hs00744661_sH MT1F	0.3	0.03918087	4	Downregulated	Downregulated
Hs00745167_sH MT1X	338	0.007835819	1	Downregulated	Downregulated
Hs00823168_g1 MT1H	281	0.15207693	Insignificant	Insignificant	Downregulated
Hs00828387_g1 MT1M	5,273,946	0.024191722	*	Upregulated **	Downregulated
Hs01591331_g1 MT1L	354	0.09650772	Insignificant	Insignificant	Downregulated
Hs01921768_s1 MT3	457	0.33829007	Insignificant	Insignificant	Insignificant
Hs01938284_g1 MT1E	307	0.035819974	3	Downregulated	Downregulated
Hs02379661_g1 MT2A	252	0.042840768	5	Downregulated	Downregulated
Hs02578922_gH MT1G	336	0.074958876	Insignificant	Insignificant	Downregulated

* No number in volcano plot image. ** The result of the expression of the MT1M isoform in the validation (upregulated) was contrary to that obtained in the first gene expression study, which showed it as downregulated.

**Table 5:** Comparison of groups by number of patients in each group and results with respect to MT genes.

Biological Group	Number of Participants	Comparative Group	MTs Results
PD+RI+	4	PD−RI−	Altered
PD+RI−	6	PD+RI+	Insignificant
PD−RI−	7	PD+RI−	Altered
PD−RI+	0	—	—

## Data Availability

The data presented in this study are available on request from the corresponding author.
